# Supplementary material for: Two Synthetic Peptides Corresponding to the Human Follicle-Stimulating Hormone β-Subunit Promoted Reproductive Functions in Mice
Source: Int J Mol Sci. 2022 Oct 3;23(19):11735. doi: 10.3390/ijms231911735 (PMC9570415; doi:10.3390/ijms231911735)
Supplement: Supplementary file 1 [file ijms-23-11735-s001.zip › ijms-1911283-supplementary.pdf]

Supplemental files:

Supplementary Table S1. Amino acid sequence of FSHβ13AA/SHβ16AA and their scrambled control peptides

Peptide	Sequence	Peptide mass percentage (%)
FSHβ13AA	LVYKDPARPKIQK	100
Scrambled FSHβ13AA mix	VYAKPLQKDPKIR	25
	DYRKAQPPKLKIV	25
	PADKKKLIRYPVQ	25
	RLAVIKQPKYKPD	25
FSHβ16AA	TRDLVYKDPARPKIQK	100
Scrambled FSHβ16AA mix	KILDPRKPTYDVKAQR	25
	KRQKPVYKRLTDDAIP	25
	PDKQLTYPVRKAIDRK	25
	YPLVKPRQTKIDKDRA	25

The mixed scrambled peptide for FSHβ13AA and FSHβ16AA were generated at Mimotopes website (http://www.mimotopes.com).

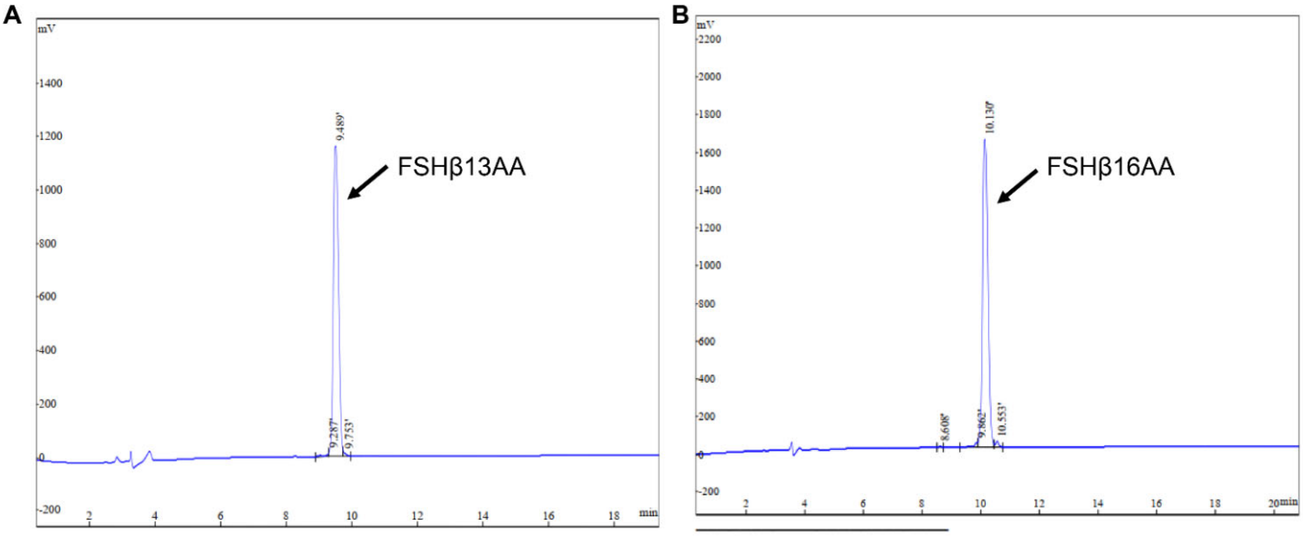

Rank	Time	Name	Conc.	Area
1	9.287		0.696	83331
2	9.489		98.58	11802714
3	9.753		0.7243	86718

Rank	Time	Name	Conc.	Area
1	8.608		0.1562	31272
2	9.862		1.02	204189
3	10.130		97.79	19577075
4	10.553		1.038	207901
Total			100	20020437

Supplementary Figure S1. High-performance liquid chromatography (HPLC) purification of FSHβ13AA and FSHβ16AA peptides. According to peak areas, the purity of FSHβ13AA and FSHβ16AA was equal to 98.58 and 97.79%, respectively.
